# Road Traffic Injury as a Major Public Health Issue in the Kingdom of Saudi Arabia: A Review

**DOI:** 10.3389/fpubh.2016.00215

**Published:** 2016-09-30

**Authors:** Erica DeNicola, Omar S. Aburizaize, Azhar Siddique, Haider Khwaja, David O. Carpenter

**Affiliations:** ^1^Institute for Health and the Environment, University at Albany, Rensselaer, NY, USA; ^2^King Abdullah University, Jeddah, Saudi Arabia; ^3^QEERI, Hamad Bin Khalifa University, Doha, Qatar; ^4^New York State Department of Health, Wadsworth Center, Albany, NY, USA; ^5^Department of Environmental Health Sciences, School of Public Health, University at Albany, Albany, NY, USA

**Keywords:** Saudi Arabia, injury, road traffic injury, traffic safety, public health

## Abstract

Injury was the largest single cause of disability-adjusted life years and death in the Kingdom of Saudi Arabia in 2013. The vast majority of injury-related fatalities are deaths caused by road traffic. Measures to control this serious public health issue, which has significant consequences for both Saudi families and the Saudi economy as a whole, have been underway for years but with little success. Most attempts at intervening revolve around attempts for enforcing stricter traffic laws and by installing automated traffic monitoring systems that will catch law breakers on camera and issue tickets and fines. While there has been much research on various factors that play a role in the high rate of road traffic injury in The Kingdom (e.g., driver behavior, animal collisions, disobeying traffic and pedestrian signals, environmental elements), virtually no attention has been given to examining *why* Saudi drivers behave the way that they do. This review provides a thorough account of the present situation in Saudi Arabia and discusses how health behavior theory can be used to gain a better understanding of driver behavior.

## Introduction

According to the Global Burden of Disease (1), injury is the leading cause of disability-adjusted life years (DALYs) lost in the Kingdom of Saudi Arabia. In 2013, the most frequent cause of death in the Kingdom of Saudi Arabia was injury (1). According to Saudi Arabia’s Ministry of Health, injury was implicated in 18.5% of deaths within the Kingdom (2). Intentional injuries (i.e., homicide and suicide) account for only a small percentage of deaths caused by injury in the Kingdom of Saudi Arabia. The homicide rate in Saudi Arabia is among the lowest when compared with other countries internationally (3). Data from the National Statistics Office indicates that the number of recorded homicides in the Kingdom of Saudi Arabia during the period between the years 1999 and 2007 ranged from 173 to 301 annually, yielding annual rates of homicide between 0.9 and 1.3 per 100,000 population. For comparison, the annual homicide rates in the United States and United Kingdom ranged from 4.8 to 5.0 and 1.5 to 2.1 per 100,000 population, respectively, during that same time (4). Of the homicides that do take place in Saudi Arabia, one study in the city of Dammam showed that over 80% of victims were males, and the majority (70%) were between the ages of 21 and 50 years old. Fatal injuries related to blunt trauma and strangling were the most common among homicide cases in this study (40%), followed by stabbing (34.4%), trauma to the chest and head (29.6 and 26.4%, respectively), and firearms (25.6%) (5).

Suicide is considered a criminal act under Islamic law, and carries stigma and condemnation. Allah makes this message explicit in the Qur’an, saying “And do not kill yourselves. Surely, Allah is Most Merciful to you.” It is, thus, not surprising that the majority (79.36%) of those who successfully commit suicide within Saudi Arabia are expatriates, not Saudi nationals (6). Analysis of data from the Forensic Medical Center in Damman collected from 2000 to 2007 showed death from suicide to occur more frequently in males (>80% of cases reviewed) than females, with an average ratio of males to females of 4.5:1 over this 7-year span (6). Nearly three-quarters of suicide cases were between the ages of 30–50 years old. Hanging was the most common method used among both males and females (6–8).

Unintentional injury is responsible for the majority of injury-related deaths in Saudi Arabia, and the majority of trauma (80–85%; both fatal and non-fatal) sustained by Saudis and non-Saudis are caused by road traffic injuries, an issue that has become increasingly prominent and severe in Saudi Arabia over the past few decades (9, 10). Studies that have reviewed death certificates from major city hospitals consistently found injuries/fractures to be the leading cause of death in individuals aged 45 years or younger, especially males (11). For example, in 2012, Alshahri et al. examined the epidemiology of traumatic spinal cord injury for all patients admitted to a Saudi hospital with traumatic spinal cord injury between 2003 and 2008 and found that 85% of patients’ traumatic spinal cord injuries were caused by road traffic collisions, followed by falling (9%), gunshot wounds (5%), and injury while participating in sport events (1%) (12). Road traffic collisions were also found to be the most common cause of traumatic head injury in children (age 12–16 years; 47.2%) and adolescents (age 16–18 years; 74.4%) in a similar study conducted in Saudi Arabia that examined cases from 2001 to 2009 (13).

In Ministry of Health hospitals, one-fifth of hospital beds are consistently occupied by road traffic injury victims who also account for 80% of the deaths that occur at these hospitals (14). A recent study by Hassan et al. compared road traffic injury data from the United States and Saudi Arabia and found that while only 2% of road traffic collisions in the US were fatal, 23% of road traffic crashes involved fatalities in Saudi Arabia (15). It has been estimated that if the traffic situation in Saudi Arabia is left unattended, it could escalate to four million crashes per year by 2030, meaning more injuries and more fatalities (16).

The societal implications of this public health issue are significant as the loss of productive young persons to injury or death has serious consequences for Saudi families and the larger society as a whole (12, 17). In 2012, 73% of the 1,100 road traffic collision victims in the city of Riyadh were under 35 years old (18), and the loss of a family member brings not only heartbreak but can also cause financial hardships and a change in household dynamics, especially if the member lost is a major source of income (9). Saudi Arabia spends roughly SR 13 billion ($6.25 billion USD) annually to treat road traffic-related injuries and on other matters related to road traffic, which is roughly 4% of the national income (16). However, the true economic cost is difficult to estimate because it includes the cost of medical care and rehabilitation services and the loss of productivity due to disability or absenteeism (9).

The discovery of oil and subsequent oil boom during the 1950s was met with a sharp rise in urban development and living standards (14, 19, 20), prompting immigration into the Kingdom and surrounding Gulf countries (21) and significant population growth in Saudi cities in a short period of time (22). As a result, road networks underwent significant changes to accommodate the growing volume of commuters (19, 20). With extremely limited public transportation and very low gas prices (23), motor vehicles became the primary mode of transportation (24) and the number of cars owned and on Saudi roads has increased exponentially over the past four decades (14, 19). This increase in the number of registered vehicles has led to unprecedented traffic congestion within the major cities (25).

Our purpose in writing this literature review is to create a thorough and comprehensive picture of the traffic safety situation in Saudi Arabia, and to identify relevant areas of information that are lacking. To accomplish this, we draw upon multiple sources that describe or analyze various aspects of traffic safety in Saudi Arabia specifically, as well as research findings from other countries in order to draw connections to concepts and theories that are relevant to the situation in Saudi Arabia. Specifically, we consider the role of social and environmental factors, traffic enforcement, driver education, and past and present intervention strategies. Additionally, we refer to public health theory as a tool for examining the individual attitudes and motivations behind driving behaviors of drivers in Saudi Arabia, a factor that has been largely ignored by researchers examining road traffic injuries in Saudi Arabia.

## Materials and Methods

Data were assembled through pubmed and Google searches (injury, Sandi Arabia, accidents, automobiles, pedestrians), internal Saudi agency documents, and from websites and newspapers. Some documents were provided by the Saudi coauthors that had been on Saudi websites and newspapers but are no longer found there.

## Results

The great majority of road traffic injuries (83.65%) between 2004 and 2009 occurred in cities (see Table 1) (26). The growth of cities mirrors the rising number of road traffic collisions since the 1970s (24), a trend that is illustrated in Table 2, which shows available data for the number of injuries and fatalities annually from 1971 to 2013. The associations between the variables in Table 2 were analyzed using Pearson correlation and the results can be found in Table 3. The results of the Pearson correlations indicate that there are statistically significant associations among all of these factors (population, number of vehicles, number of collisions, number of injuries, and number of fatalities).

**Table 1:** **Location of vehicular injuries (inside vs. outside city limits) in Saudi Arabia, 2004–2009. Data from Ref. (26)**.

Year	Number of injuries inside the city	City population (in million)^a^	Injuries per 10,000 person, inside the city	Number of injuries outside the city	Population of outside the city (in million)	Injuries per 10,000 person, outside the city	Total population (in million)^b^
2004	230,463	4.09	5.63	62,818	19.75	0.32	23.84
2005	234,947	4.22	5.57	61,068	20.47	0.30	24.69
2006	231,267	4.35	5.32	52,351	21.02	0.25	25.37
2007	373,102	4.53	8.24	62,162	21.39	0.29	25.92
2008	420,616	4.63	9.08	65,315	21.74	0.30	26.37
2009	415,910	4.87	8.54	68,895	21.97	0.31	26.84
Percentage	83.65			16.35			

^a^Data retrieved from Central Department of Statistics and Information, Saudi Open Data. Available from: http://www.data.gov.sa/central-department-statistics-and-information

^b^Data retrieved from UN-ESCWA (n.d.) website: http://www.escwa.un.org/popin/members/SaudiArabia.pdf

**Table 2:** **Road traffic injuries in Saudi Arabia, 1971–2013**.

Year	Population in millions	Number of registered vehicles	Number of collisions	Number of injuries	Number of fatalities	Severity rate (SR)
1971	6.25	144,768	4,147	5,483	570	1.46
1972	6.50	180,185	7,197	6,530	834	1.02
1973	6.75	242,974	9,808	7,901	1,058	0.91
1974	6.85	355,022	10,897	8,771	1,154	0.91
1975	7.38	514,361	13,475	10,532	1,594	0.90
1976	7.90	774,443	15,709	11,606	1,975	0.86
1977	8.43	1,112,973	15,785	11,413	2,033	0.85
1978	8.96	1,432,909	18,051	14,824	2,378	0.95
1979	9.48	1,723,116	17,743	16,832	2,871	1.11
1980	10.01	2,069,479	18,758	16,218	2,731	1.01
1981	10.54	2,467,903	17,897	15,872	2,427	1.02
1982	11.06	3,018,811	21,597	18,616	2,953	1.00
1983	11.59	3,569,009	24,594	21,475	3,499	1.02
1984	12.12	3,919,871	27,348	21,850	3,338	0.92
1985	12.65	4,144,245	29,052	22,630	3,276	0.89
1986	13.17	4,280,986	32,092	22,602	2,703	0.79
1987	13.69	4,427,991	32,024	23,723	2,814	0.83
1988	14.22	4,574,244	32,584	23,059	2,585	0.79
1989	14.75	4,767,922	35,744	23,278	2,647	0.73
1990	15.27	4,950,466	35,799	23,526	2,697	0.73
1991	15.80	5,117,441	37,127	25,516	3,232	0.77
1992	16.33	5,328,505	40,076	27,385	3,495	0.77
1993	16.85	5,588,013	85,277	34,880	3,719	0.45
1994	17.38	5,861,614	125,324	32,133	4,077	0.29
1995	18.57^c^	–	130,544	28,606	3,604	0.25
1996	–	–	135,763	25,078	3,131	0.21
1997	–	–	153,727	28,144	3,474	0.21
1998	–	–	264,326	31,059	4,290	0.13
1999	–	–	267,772	32,361	4,848	0.14
2000	20.15^c^	–	131,876	28,998	4,419	0.25
2001	–	–	131,876	28,379	3,913	0.24
2002	–	–	223,816	28,372	4,161	0.15
2003	–	–	261,872	30,439	4,293	0.13
2004	23.84^c^	–	293,281	34,811	5,168	0.14
2005	24.6^c^	–	296,015	34,441	5,982	0.14
2006	25.37^c^	–	283,648	35,884	5,883	0.15
2007	25.92^c^	–	435,264	36,025	6,358	0.10
2008	26.37^c^	–	485,931	36,489	6,458	0.09
2009	26.84^c^	–	484,805	34,602	6,142	0.08
2010	27.26^c^	6,599,216^a^	498,203	38,595	6,596	0.09
2011	–	–	544,179	40,000	7,153	0.09
2012	–	–	–	–	–	–
2013	29.2^b^	8,000,000^a^	–	–	–	–

*^a^Data from WHO Global Health Observatory Data Repository (2013)*.

*^b^Data retrieved from World Population Statistics (2013)*.

*Data obtained from Dr. Aburizaize’s personal communication with Motor Vehicle Department of KSA, 2014 May*.

^c^Data retrieved from UN-ESCWA (n.d.) website: http://www.escwa.un.org/popin/members/SaudiArabia.pdf

**Table 3:** **Correlations among the different variables from Table 2**.

		Year	Population	Number of vehicles	Number of collisions	Number of injuries	Number of fatalities	SR
Year	Pearson correlation	1						
	*N*	41						
Population	Pearson correlation	0.998^a^	1					
	*N*	24	24					
Number of vehicles	Pearson correlation	0.989^a^	0.990^a^	1				
	*N*	24	24	24				
Number of accidents	Pearson correlation	0.878^a^	0.776^a^	0.731^a^	1			
	*N*	40	24	24	40			
Number of injuries	Pearson correlation	0.960^a^	0.972^a^	0.968^a^	0.802^a^	1		
	*N*	40	24	24	40	40		
Number of fatalities	Pearson correlation	0.940^a^	0.848^a^	0.863^a^	0.912^a^	0.935^a^	1	
	*N*	40	24	24	40	40	40	
SR	Pearson correlation	0.933^a^	0.734^a^	0.682^a^	0.840^a^	0.872^a^	0.841^a^	1
	*N*	40	24	24	40	40	40	40

*^a^Correlation significant at the 0.01 level (two-tailed)*.

*N = number of data points*.

The statistics commonly cited in the literature regarding the frequency of injuries and deaths due to road traffic collisions report approximately four injuries and one fatality every hour (or 20 deaths per day) in Saudi Arabia (14, 17, 27–29). However, an article published in December 2013 on *Arab News* claims that these numbers are inaccurate; according to the regional director of research on injuries at King Abdullah International Medical Research Center, Saud Al-Turki, “Death statistics cited by the traffic department only count people who die at vehicular collision scenes. It does not take into account deaths inside hospital operating theaters or intensive care units…the recorded death toll here represents only 48% of the total.” (17). This statement implies that there are actually 41 deaths due to road traffic collisions each day rather than the typically reported 20 deaths, placing Saudi roads atop the list for the most dangerous roadways globally (30). Al-Turki’s accusation of inaccuracy in police reports is supported by research that showed traffic death rates reported by police were inconsistent with traffic death rates based on death registration data, which was nearly twice as high (31).

### Causes of Road Traffic Collisions

In the *Saudi Arabia 2013 Crime and Safety Report* published by the Overseas Security Advisory Council (32) of the United States Department of State, potential visitors to Saudi Arabia are cautioned about the high frequency of road traffic collisions and how they often “result in serious injuries and/or fatalities.” It goes on to explain that

Speeding is common, especially by owners of high-end sport cars and luxury vehicles. ‘Stop’ and ‘Yield’ signs are often ignored. Drivers pass at any time from any direction, and turn signals are rarely used. Passing on blind curves from both directions is also common. Pedestrians and livestock in the road can be a hazard; in some cases, shepherds have bedded their sheep near major highways at night, resulting in collisions between vehicles and livestock that stray onto the road. Motorists should drive defensively, use extreme caution, and wear seatbelts at all times.

Speeding, disobeying traffic signals, sudden lane change, and driver errors are frequent causes of road traffic collisions (15, 17, 24, 33). One study found that among young drivers who had been involved in road traffic collisions, high speed was the primary cause of these events and that they had been traveling ~81 km/h (50 mph) within city limits and 127 km/h (80 mph) outside of the city (17). The urban speed limit in Saudi Arabia is typically 45 km/h (~30mph) and ranges from 80 to 120 km/h (~50 to 75 mph) on highways (23). Time constraint is a very common reason for speeding reported by drivers, and many feel road congestion contributes to this (22). Most severe crashes occur at non-intersection locations (about 97%) versus within intersections, and the odds of involvement in a fatal crash is 1.137 times greater at non-intersection locations (15). Overtaking other cars from the wrong side is another example of driver error that often leads to road traffic collisions (24), along with tire blowouts due to improperly maintained tires (34). Table 4 provides statistics on the number of road traffic collisions by their causes in Saudi Arabia from 2004 to 2011 (26). Table 5 shows the regional distribution of causes of road traffic collision in the year 2011 (35).

**Table 4:** **Number and percent of collisions by their causes in Saudi Arabia, 2004–2011**.

Year	High speed	Improper turning	Improper passing	Violation of traffic signal	Improper stopping	Driving under the influence of drugs	Other reasons
2004	99,602	30,539	27,001	13,861	26,432	325	95,521
2005	91,057	23,451	25,478	13,853	22,496	159	119,521
2006	79,981	22,510	17,638	12,544	21,502	124	129,349
2007	152,868	31,589	26,469	15,869	25,808	134	120,189
2008	220,556	37,059	43,719	28,497	41,869	255	123,267
2009	222,429	35,881	29,225	49,785	30,828	131	116,401
2010	165,963	37,889	33,963	66,503	37,327	118	156,440
2011	134,108	56,664	58,384	56,717	48,955	1,696	187,655
Percentage	35.69	8.43	8.01	7.88	7.81	0.09	32.08

**Table 5:** **The regional distribution of traffic collisions occurred for the year 2011**.

Area	Over speeding	Irregular rotation	Irregular bypass	Uncomely with traffic signal	Irregular stop	Other reasons	Total	% in ^a^KSA
Riyadh	40,140	23,569	28,111	44,924	18,062	8,991	163,797	30
Western region	8,913	11,726	4,443	1,092	4,674	91,958	122,806	23
Eastern region	31,498	6,466	8,772	4,847	8,978	40,179	100,740	19
Rest of the ^a^KSA	53,557	14,903	17,058	5,854	17,241	48,223	156,836	29
Total	134,108	56,664	58,384	56,717	48,955	189,351	544,179	100
%	25	10	11	10	9	35	100	

*^a^Kingdom of Saudi Arabia*.

#### Social Factors

Research has consistently found an association between driver gender and age and risky driving behavior such that males and younger persons are more likely to engage in driving behaviors that lead to collisions (36–38). Women are prohibited from driving in Saudi Arabia (39), which means the roads are more highly populated with male drivers than in most other countries. The median age of the Saudi population is 25.7 years (26.7 years for men and 24.4 years for women) (40), indicating that the population of drivers is skewed toward the younger age range that is more likely to engage in risky driving behaviors (38). An article published in November of 2013 from *Arab News* reported 2,000 cases of drivers running red lights within a span of 2 weeks in the city of Riyadh, mostly by teenagers (17). According to Deery, young novice drivers “play a disproportionately large role in traffic crashes” because of their age and inexperience [(36), p.225]. For young people, driving represents autonomy, adulthood, status, and provides an opportunity to carry out risky behaviors. Novice drivers may not have the driving skills and ability needed to properly assess the hazards of a driving situation to accommodate their risky driving styles (36). Drivers often engage in thrill seeking and are overconfident about their driving abilities. Furthermore, drivers commonly believe that they always have the right of way, and do not practice common courtesy while on the road or yield to other drivers (9).

Another social factor associated with the rise in road traffic injuries in Saudi Arabia is immigration, as expatriates from other countries are often unfamiliar with local driving requirements and conditions and practice different driving habits (24). Non-Saudis are accountable for nearly 40% of road traffic collisions in Saudi Arabia (9).

Driving under the influence of alcohol is a common factor in collisions around the world, accounting for one-third of vehicle-related events in the United States (41). However, alcohol consumption and drug use is illegal in Saudi Arabia and, therefore, is not a common factor for road traffic collisions (14). According to the statistics in Table 4 (obtained from the General Department of Traffic at the Ministry of Interior in Saudi Arabia), it is estimated that ~0.09% of road traffic injuries occurring from 2004 to 2011 were caused by drivers driving under the influence of drugs (26). However, measuring the true impact of substance use while driving in Saudi Arabia is extremely difficult because of the high likelihood of reporting bias (12).

#### Electronic Devices

The information about mobile phone use while driving in Saudi Arabia is inconsistent. Some reports state that the use of all electronic devices is illegal (23, 42), while others claim that there are presently no laws limiting mobile phone use while driving, and that many individuals use these devices while driving (43). According to a report from the United Nations in 2013, Saudi Arabia has the greatest proportion of mobile phone users worldwide at 188% (44), which means, at the very least, that most (if not all) drivers will have a mobile phone at their disposal while driving a car. Studies have shown mobile phone use while driving to be a contributing factor to road traffic injuries in Saudi Arabia (9).

The legality of mobile phone use while driving is an important issue to clarify and address, as a study examining the traffic safety knowledge and compliance among youth (high school and university students) found that 85% of study participants reported using mobile phones while driving (27). Another study by Osuagwu et al. (43) found that making or receiving phone calls while driving was accompanied by seven times greater relative risk of road traffic injury involvement among drivers in Saudi Arabia than those who did not.

#### Environmental Factors

Thirty-nine percent of injuries in the Kingdom of Saudi Arabia are due to tire blowouts caused by extreme heat (9, 17) and improperly maintained tires that cannot withstand these conditions (34). Extreme heat also reduces the mental capacity of drivers via heightened levels of stress. Other environmental factors, such as rain, fog, and dust do not play a significant role in road traffic collision in the Kingdom of Saudi Arabia (9).

The layout of road networks also plays a role in road traffic collisions. Some authors have described four-lane roads that immediately merge into three lanes after an intersection, or closures of highway entrances and exits during rush-hour due to construction (9). Roundabouts are common, and instead of improving the flow of traffic through intersections as intended, many drivers do not follow roundabout regulations (i.e., signaling to exit the roundabout, driving in the proper lane, yielding to other drivers already within the roundabout) so a high frequency of road traffic injuries occur in these intersections (20).

Collisions with camels are also commonplace within the Kingdom and – as with other animal–vehicle collisions with large animals – the outcome is generally bleak (45). Over 600 camel–vehicle collisions occur every year, causing major property damage and numerous deaths. Most of these collisions occur on straight sections of rural roads and at night (46). As mentioned previously, livestock bedded near highways by shepherds also pose a hazard for drivers if sheep wander onto the road (32).

#### Pedestrian-Related Crashes

Previous studies have found that over three-quarters of pedestrians involved in road traffic collisions were hit while crossing a road without utilizing a crosswalk. Head and neck injuries resulting from these collisions accounted for 34% of fatalities (15). Children are more often injured as pedestrians than as vehicle passengers (47), often as unsupervised children playing in the streets who then fall victim to oncoming traffic (9). Many children are killed or suffer long-term neurological damage from head trauma (47). One study found that nearly 26% of crashes in Saudi Arabia were pedestrian related. For comparison, only 7% of injuries are related to pedestrians in the US (15). In one study that investigated driver attitudes in the city of Tabuk, only 28 to 41% of respondents felt that pedestrian (and cyclist) safety was a moderate to serious issue when considering it in terms of the general public; however levels of concern rose to 61–69% when respondents applied the situation to themselves (22).

### Traffic Law Enforcement

The Saher system was introduced as a tool for decreasing the incidence of road traffic injuries in Saudi Arabia. It is “an automated traffic control and management system” that uses a series of digital cameras placed along the roadside that can measure the speed of cars, capture photos of erring vehicles, recognize repeat offenders, issue tickets and notifications of fines for traffic violations, alert police patrols to the location of a collision, and monitor and control traffic lights automatically in order to improve the flow of traffic through intersections (48). According to the regional director of research on injuries at King Abdullah International Medical Research Center, Saud Al-Turki, the number of accidents and injuries were reduced during the first decade Saher was in use, but there has not been a decline in the number of deaths (17). Al-Turki says that the Saher system focuses on speeding and running red lights, which only account for 31% of road traffic collisions (17). Because the system has not been implemented in a more comprehensive way, traffic officials are said to ignore the other 69% of traffic violations that lead to road traffic collisions – like reckless driving – which encourages drivers to continue breaking the traffic rules that are not being enforced (30).

Research on traffic law enforcement suggests that certainty of punishment, rather than severity of punishment, is key in deterrence (49). According to the Ministry of Interior, there are predetermined punishments that drivers will face if caught violating traffic laws (see Table 6), but if the laws are not strictly enforced and punishments for violations are inconsistently applied, simply having a guideline for penalties in place is not an effective deterrence. For example, the previously mentioned article from *Arab News* reporting the large number of cases of running red lights in Riyadh found that out of the 2,000 violators, only 200 motorists were arrested, detained, and fined. Additionally, Hassan et al. (50) showed that the majority (60%) of crashes in the city of Riyadh occur at night, when enforcement tends to be more lax, and rule breaking in roundabouts has been blamed on poor police enforcement at intersections (20). This evidence is supported by a study of drivers in Northern Kosovo and Serbia in which Stanojević et al. (51) found that poor traffic law enforcement led to increased speeding behaviors in drivers, and that longer periods without enforcement also led drivers to take on more positive attitudes toward speeding.

**Table 6:** **Traffic violations and penalties as reported by the Ministry of Interior, Kingdom of Saudi Arabia**.

Level of violation	Penalty	Examples
Category I	A fine of at least SR 500 and no more than SR 900, may also keep vehicle in custody	Driving without a driving license Driving a vehicle without plates (one or both missing) Using forged registration plates Not using headlights when dark or while driving in poor weather conditions Not stopping for a red light Moving fast, recklessly between vehicles on public roads Driving in the opposite direction Overtaking vehicles in curves and uphill Exceeding the speed limit by more than 25 km/h Violating traffic signs (including police direction)
Category II	A fine of at least SR 300 and no more than SR 500, may also keep vehicle in custody	Not complying with road regulations at intersections Leaving objects in the road that endanger others Using an expired license Overtaking school buses while loading or unloading Carrying excess passengers than can fit in car Tampering with traffic signs Exceeding speed limit by 25 km/h
Category III	A fine of at least SR 150 and no more than SR 300	Failure to have vehicle regularly inspected Driving without carrying a license or vehicle registration Not wearing seatbelts Not using safety belts for children Using a cell phone while driving Misusing vehicle’s horn Not following regulations for equipping trailers Violating rules for driving on roads Not wearing a helmet while riding a motorbike Letting domesticated animals onto roads Driving in lanes not intended for driving Using unauthorized devices inside vehicle

Category IIII	A fine of at least SR 100 and no more than SR 150	Fixing logos or posters to vehicle that contradict public morals Throwing objects outside of moving vehicle Pedestrians crossing in undesignated places Pedestrian non-compliance with pedestrian signals Not focusing on road while driving Not having an insurance policy Parking in spaces designated for people with special needs when you do not have special needs Leaving vehicles in unauthorized areas on public roads unnecessarily

In addition to consistency of punishment, trust in authority is another important factor in deterrence. Notice in Table 6 that the penalties for all categories of traffic violations are primarily or solely monetary. Table 7 shows that these monetary fines are actually very modest. Research has shown that people are less inclined to trust authority and comply with traffic enforcement if they perceive it as being used as a tool for acquiring money rather than reducing harm (49). A study by Alhindi and Albawardy (28) found that the majority of drivers perceive the Saher system’s objectives to be collecting traffic fines (62.6%) and/or reducing collisions (56%). Only 37% of drivers felt preserving lives was an objective of Saher.

**Table 7:** **Penalties for violating Kingdom of Saudi Arabia traffic laws**.

Violation	1st offense penalty		Repeated offense penalty	
	Fine (SR)	Days in jail	Fine (SR)	Days in jail
Running a red light	900	3	1,800	
Wrong way on one-way street	900	3	1,800	6
Speeding	900	3	1,500	6
Parking in a non-parking area	500	3	Same as 1st offense	
Parking on a side walk	500	3	Same as 1st offense	
Failure to carry driver’s license	300	NIL	Same as 1st offense	
Driving with an expired driver’s license	300	NIL	Same as 1st offense	
Driving without a valid driver’s license	900	NIL	Same as 1st offense	
Expired vehicle registration	500	3	Same as 1st offense	
Passing in a no passing zone	900	3	Same as 1st offense	
Leaving the scene of an accident	900	3	Same as 1st offense	
Failure to stop for police	300	3	Same as 1st offense	
Reckless driving	1,500	20 plus 20 lashes	3,000	20 plus 20 lashes and vehicle confiscation
Accompanying a reckless driver	1,500	20 plus 20 lashes	3,000	20 plus 20 lashes

Adapted from table retrieved from http://udhailiyah.com/wpcontent/uploads/2008/01/police-ksa-traffic-law-w-e-f-1-1-1429h-2.pdf

Nagin [1998 cited by McKenna (49)] argues that effective deterrence from unwanted behavior comes from sanctions that are socially isolating or stigmatic to the person being punished, and that this cannot be the case if a given punishment is “commonplace” (49). McKenna [(49), p. 214] illustrates this point using speeding behaviors, suggesting that “As long as speeding is commonplace, any punishment will not be socially isolating”. He goes on to hypothesize that if driver education courses were able to reduce the number of people speeding on the roads and, thus, the number of people punished for speeding, this may create a scenario where receiving sanctions for speeding does become socially isolating (49). This could dissuade even more individuals from speeding for fear of being caught and stigmatized. According to the official Saher website, 9 million traffic violations occur annually (52). Assuming this figure is accurate, and with ~8 million registered vehicles in Saudi Arabia as of 2013 (53), this means that, statistically speaking, every vehicle would be involved in an average of 1.125 traffic violations each year. With traffic violations so prevalent, it is unlikely that violators feel stigmatized when caught and punished for more common violations like disobeying traffic signals or speeding, especially when most of these violations are detected and penalties are issued covertly by an automated system.

To reduce the number of injuries and deaths due to road traffic collisions, many attempts at imposing stricter seatbelt laws have been made. Seat belts lessen the severity of injuries experienced by vehicle drivers and passengers by keeping them from hitting objects around them or being ejected from the vehicle upon impact (19), and it has been estimated that if all drivers and passengers used seatbelts, 27% of road traffic injury fatalities could have been avoided (17). In 2000, Saudi Arabia passed laws requiring all drivers and front seat passengers to use seat belts (19). Despite these laws, little impact has been observed. A study of two Riyadh suburbs shortly after the stricter seatbelt laws were imposed showed that on average, only 60% of drivers and 22.7% of front seat passengers reported using seatbelts while driving (19, 21). Even with this improvement, these statistics are still considered low compared to seat belt use rates in other developed countries, which typically stand around 80% (19). Seat belt compliance in Saudi Arabia remains very low (12) especially with the growing leniency in enforcement by Saudi police since the implementation of seat belt law (54).

### Driver Education

In Saudi Arabia, private driver-training programs offer driver education that consists of lectures on traffic laws and regulations, on-road training, and driving simulations. All applicants for driving licenses are required to complete the lecture course at minimum in order to obtain a driving license even if they have learned to drive elsewhere, as long as such a course is available in their particular area. In areas where driver education programs do not exist, young drivers are taught by friends or relatives (55).

Our search of the literature yielded only a single paper analyzing the effectiveness or impact of driver education programs in Saudi Arabia. In his paper from 1993, Al-Subhi (55) evaluated the effectiveness of formal driver education in preventing road traffic collisions and found no significant difference in the number of road traffic collisions reported between drivers with or without formal driving education. Al-Subhi suggested that these findings could be indicative of a failed system for driver education in Saudi Arabia or that risky driving practices are part of the Saudi cultural pattern, but also pointed out that his findings could have been confounded by the sample used – which included only less experienced, college-age drivers – limiting the ability to draw any generalizable conclusion based on the results. This study did, however, make it clear that knowledge of traffic safety rules and regulations among the study population (young males) was poor.

Despite the potential limitations of Al-Subhi’s study, a review of the international literature on driver education support his findings on the basis that, overall, researchers have not found driver education to be an effective countermeasure for road traffic collisions (56). To our knowledge, there has not been another study evaluating driver education programs in Saudi Arabia. A study conducted in 2011 evaluating traffic safety knowledge indicated that most of the participants (15–19 year olds) were knowledgeable about dangerous driving practices and what driving behaviors (i.e., speeding, lack of attention, non-compliance with traffic safety regulations, stunts, and lack of experience) contribute to most collisions. Many, however, did not know important traffic regulations and signs for yielding to traffic and pedestrians, which the researchers believe is a major contributor to the high incidence of traffic collisions (27). More than a decade later, with road traffic collisions now the top cause of death, additional evaluations of the driver education system may be beneficial to inform traffic officials about the present state of the system. However, it seems unlikely that replicating Al-Subhi’s study would yield different results.

### Interventions

The year 2011 marked the launch of the Decade of Action for Road Safety by the World Health Organization (WHO) to improve road and vehicle safety, promote safer driving behaviors, and expand emergency services with the goal of saving the lives of millions (42). Actions taken in Saudi Arabia have included providing education through media about the dangers and consequences of reckless driving, along with the expanded utilization of speed cameras (Saher) on roadways to control high speed (17). At the end of 2013 the Council of Ministers approved a National Strategic Plan for Traffic Safety that aims to reduce the number of traffic injuries by 30% over the 10 years following its implementation and Dr. Abdulaziz Khoja.

Minister of Culture and Information stated “the Strategic Plan aims to draw a national traffic safety policy outlining broad future directions for traffic safety system in the kingdom. The plan will be built on a number of strategies including developing a full-fledged and comprehensive system for urban and transport planning” (16).

Saudi Aramco, officially the Saudi Arabian Oil Company founded in 1933, has taken action in recent years to address what they call “appalling” statistics in terms of fatality rates on Saudi streets. To address this issue, Abdulaziz F. Al-Khayyal, senior vice president of Industrial Relations at the company, appointed a team to run “The Traffic Safety Signature Program” which encourages all citizens of Saudi Arabia to “become advocates for traffic safety … to spread the word about traffic safety to family and friends” (57). The foundation of their efforts in promoting traffic safety in Saudi Arabia utilizes the “Four E’s” of traffic safety, which include Engineering, Education, Enforcement, and handling Emergencies. Table 8 provides a brief outline of how each “E” is being addressed. In addition to the actions outlined in Table 8, Saudi Aramco Medical Services, Transportation, Loss Prevention, Training and Development, Industrial Security, and Personal employees have given presentations throughout the Kingdom about the importance of the traffic safety, have been taken to parking lots to inspect tires and leave warnings on vehicles with poor tires to alert the owner, and have been handing out pamphlets with traffic safety information in Saudi communities.

**Table 8:** **The four E’s of the Traffic Safety Signature Program**.

Four E’s	Action
Engineering	Redoubled efforts with Ministry of Transportation to raise standards for improved and safer designs for roadways Conducted many studies on traffic-flow and engineering on highways
Education	Provide access to company’s portable hands-on driving simulators to both young and adult drivers Created the first comprehensive driver-training manual in the Middle East – now used in Saudi public schools Encourage everyone to engage in conversation about traffic safety
Enforcement	Crack down on speeders and reckless drivers by Traffic Police and Saudi Aramco Industrial Security
Emergency care	Teaching Eastern Province first responders latest injury triage and life support techniques for critical period between injury and transport to a hospital

Organizations, such as Saher and Saudi Aramco, have also attempted to use the media to curb reckless driver behavior. On the Saher website (58), under “Press Camping,” there are a series of posters with the mug shots of traffic law violators, followed by series of posters with images of collisions in progress and the aftermath – images of a screaming boy leaning over the bloodied body of his friend before and after the paramedics arrived, a picture of a child in a stretcher, and even an image of a wreck as a man’s head breaks through the sunroof of his overturning car. It is unclear whether these images are real or staged. A video public service announcement titled “Wrecking Lives” by Saudi Aramco [(59), from 2013] available on YouTube shows a Saudi man talking about the impact of road traffic accidents on the thousands of families who lose their loved ones saying, “Inside every splintered wreck was someone’s Father, Mother, Child” as wrecked cars fall out of the sky around him. He asks viewers, “When next you drive, will there be more lives ruined? Or will you help make the numbers drop?”. It appears that these campaigns seek to show drivers what tragedy could await them if they do not practice safe driving and are involved in a collision. Research has shown that these “threat-based” road safety messages or “mortality-related threat appeals” often provoke defensive reactions; for example, some individuals, particularly those with high driving self-esteem, may adopt the cognitive bias “it will never happen to me” and actually *increase* their risky driving behavior after exposure to messages of this nature (38). Given that “overconfidence” is a trait said to be common among drivers in Saudi Arabia (9), it may be wise to examine the effects of these campaigns on drivers within the Kingdom.

## Discussion

The importance of developing and implementing effective strategies for reducing the number of injuries due to road traffic collisions in Saudi Arabia cannot be overstated. Unfortunately, this issue is deceptively complex, and many attempts at reducing the incidence of road traffic collisions simply have not had significant or lasting effects. New strategies and movements, such as those from WHO, the Council of Ministers, and Saudi Aramco are still in the early stages of development and or implementation, so the success of present efforts remains to be seen.

Recommendations from the literature for addressing traffic safety suggest firm punishment for violations, stricter seatbelt laws, improving traffic collision data (18), development and implementation of a trauma system (9), providing better driver education and training for highway patrol personnel (60), improving citizens’ perceptions of the speed camera system (Saher) and changing the location of cameras based on accident location frequency (28), and/or launching massive traffic awareness programs to promote self-discipline among drivers (54). Based on their research, Hassan and Al-Faleh (50) recommend that Saudi traffic safety agencies focus their energy on non-intersection locations (where the vast majority of collisions take place), and that future traffic safety campaigns and education efforts should stress the severe consequences of speeding, abrupt lane change, and being distracted while driving, which increase the size and severity of road traffic collisions.

While improving education, traffic safety awareness, traffic and seatbelt law enforcement, collision data collection, and road, violation detection, emergency, and transportation systems are all important pieces in the effort to reduce road traffic collisions, it appears that a crucial factor has been overlooked – individual behavior, which has been identified as the “most important and most difficult factor to control” [(9), p. 50]. Certainly, the behaviors of drivers have been described and attention has been given to raising people’s awareness of the consequences of their poor driving behavior, and efforts have been made to teach better behaviors; but *why* drivers in Saudi Arabia behave the way they do and *what* motivates them to comply or not comply with traffic laws seems to have been ignored by officials and researchers alike. Plenty of research has been done elsewhere from which inferences can be drawn, similar to what we are doing in this paper; however, Saudi Arabia is a unique culture and findings from studies in other countries – mainly the US and European countries – may not be wholly generalizable to the specific situation and cultural context of Saudi Arabia.

### Understanding Driving Behaviors

In an article published in the *International Journal of Injury Control and Safety Promotion*, Al Turki [(17), p.3] identified the greatest weakness for current road safety interventions and policy in Saudi Arabia as “the difficulty in changing the behavior of adolescent drivers between 17 and 21 years, who are most likely to drive too fast and can create many accidents as a result.” This sentiment is echoed by Emad Dughaither, the manager of Saudi Aramco’s “The Traffic Safety Signature Program” who, despite all the efforts being made by this program, still feels “The biggest question that remains is how do you change behavior?” He also went on to say “Our biggest challenge yet is how to get people to take the initiative to get involved – how do we make those we care for realize that it is not somebody else’s problem, but that it is your problem and my problem and our problem?” (57). Positive movements toward better driver education, traffic safety awareness, and road system enhancement are already underway, but ultimately, these advancements may not have much impact on the individual behaviors and attitudes of risky drivers.

In order to answer the question of *how* to change behavior and thereby develop effective interventions, a greater understanding of what is driving the behavior is imperative. Also, in addition to changing existing unwanted behaviors, it is equally important to focus on *preventing* new and future drivers from adopting unwanted driving behaviors. Researchers interested in the psychology of risky driving behavior in other countries have frequently turned to public health theory to help explain why people engage in such behaviors, specifically the theory of reasoned action and planned behavior; to our knowledge, this theory has not yet been applied to the driving situation in Saudi Arabia.

**Figure 1:**
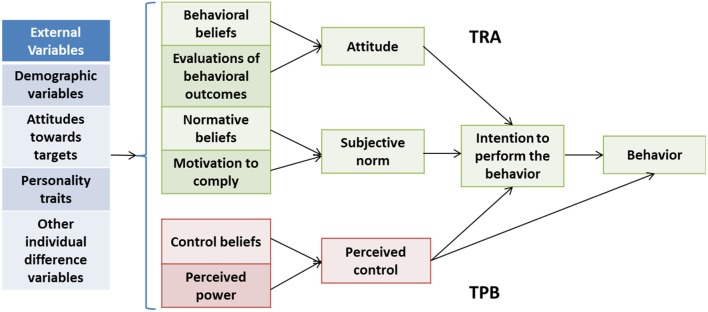
**The theory of reasoned action and planned behavior**. Revised from *Health behavior and health education: Theory, research, and practice* [(65), 4th ed., pp. 67–96].

The theory of reasoned action and planned behavior from Ajzen (61) is the extended version of the original reasoned action theory from Fishbein and Ajzen 1975 (62) (see Figure 1). The theory of reasoned action and planned behavior asserts that behavioral intention is the most robust predictor of behavior, and this relationship is moderated by an individual’s attitude, subjective norms, and perceived behavioral control relating to that behavior (37, 61). Attitude refers to the psychological tendency toward favorable or unfavorable evaluations of a particular entity (63). For example, many researchers have found a common motivator for speeding behavior among male drivers in particular (regardless of driving experience) is thrill seeking (38, 63) that reflects a positive attitude toward speeding, perceiving this behavior to be exhilarating. Indeed, Iversen (64) found that drivers’ favorable attitudes toward reckless driving behaviors (i.e., speeding) were predictive of future risky driving behavior and accident involvement.

Subjective norms correspond to “perceived social pressures from significant others or from a personal reference group” [(38), p. 1227]. Multiple studies have implicated subjective social norms as the one of the main contributors to speeding behaviors, particularly among novice drivers (63). For example, one study showed that young male drivers tend to hold biased beliefs that other drivers speed more than they actually do, and reported feeling peer-pressured to speed (66). The results from another study suggest that even thrill seeking behavior “is more influenced by external social influences, such as the normative influence of friends” [(38), p. 1233]. Perceived behavior control is “the extent to which an individual believes (i.e., is confident) that the performance or non-performance of a specific action resides within his/her volitional control or capability” [(38), p. 1227]. Elliot et al. (67) found perceived behavioral control to be the greatest predictor of speeding in their study of drivers in United Kingdom. Perceived control may be of great importance in Saudi Arabia as well, as drivers in Saudi Arabia are said to be “overconfident” in their driving ability (9), which implies strong beliefs that they are in control over their driving behavior, both in their choice to engage in the behavior and their ability to carry it out successfully.

A very important aspect of the theory of reasoned action and planned behavior is that it assumes that people are “rational actors” with underlying reasons (i.e., behavioral, normative, and control beliefs) that motivate them to perform a behavior, and that these reasons need not be related to any objective standard of rationality, logic, or correctness (65). Furthermore, Montaño and Kaspryzk [(65), p. 76] explain that

A strength of the theory of reasoned action and planned behavior is that [it provides] a framework to discern those reasons and to decipher individuals’ actions by identifying, measuring, and combining beliefs relevant to individuals or groups, allowing us to understand their own reasons that motivate the behavior of interest…Interventions can then be designed to target and change these beliefs or the value placed on them, thereby affecting attitude, subjective norm, or perceived control and leading to changes in intentions and behaviors.

Using the theory of reasoned action and planned behavior as a framework for researching and understanding the reasons and beliefs that motivate drivers in Saudi Arabia to engage in risky and reckless driving behaviors could provide a stronger, more well-informed foundation for developing intervention and educational strategies to improve traffic safety. Changing beliefs and attitudes at the individual level, and thereby individual behavior, could be the key to significant improvement in traffic safety in Saudi Arabia.

The purpose of this review was to create as comprehensive a picture of the traffic safety issue in Saudi Arabia as possible, in order to draw attention to the severity of the issue and gaps in the information that is presently available. We have found that the available data and information communicated through various avenues and forms of media to be incongruent: Saudi official personnel have contested commonly reported statistics; there is no single source of data for accident, injury, and fatality statistics from the time of Saudi Arabia’s economic boom to the present; some sources claim that mobile phone use while driving is legal, while others say it is not; and there is disagreement on the functionality of the Saher system. Furthermore, although the Ministry of Interior discloses the penalties drivers should expect for different traffic violations, another document from Kettaneh Construction (57) outlines completely different penalties for traffic violations (see Table 7). These inconsistencies suggest a need for greater transparency from Saudi traffic officials and better methods for collecting and disseminating available data and information to the public. The availability of different information and misinformation creates the potential for citizens to have an unclear knowledge or understanding of their country’s traffic safety issues and policies.

Utilizing public health theory, such as the theory of reasoned action and planned behavior as a framework for guiding research and designing interventions, is an important strategy to consider for reducing road traffic injuries. While improving road systems, developing and improving public transportation, enforcing traffic laws and issuing penalties with greater consistency, raising awareness of traffic safety, and providing education to drivers are all important pieces to a comprehensive plan of action, authors such as Al-Turki and Dughaither are spot-on in also calling attention to individual behavior, and asking the question of how to change it (17). Understanding why a behavior is performed and the motivation behind it is the first necessary step in answering that question, and the theory of reasoned action and planned behavior provides a guided tool for doing so. The theory of reasoned action and planned behavior does not, however, provide a guideline for actually changing the behavior, which is where other relevant public health theories and models of behavior change would be required to guide an intervention.

## Conclusion

With the projected annual rate of 4 million road traffic collisions per year by 2030, there is an urgent need to take robust and pointed action to develop effective and comprehensive solutions to the traffic safety problem in Saudi Arabia. The impacts of road traffic injuries on society and families who lose loved ones are severe, and a collective effort is needed to continue to understand what factors contribute to the high frequency of road injuries. Collaboration between government agencies and major companies, such as Saudi Aramco, is extremely positive, however, there needs to be greater effort and collaboration coming from Saudi citizens as well.

## Author Contributions

All authors have equally contributed to the development of the manuscript.

## Conflict of Interest Statement

The authors declare that the research was conducted in the absence of any commercial or financial relationships that could be construed as a potential conflict of interest.
